# Just-in-case medication use by ambulance paramedics responding to end-of-life care in the community: protocol for a multi-method study (RELIEF)

**DOI:** 10.29045/14784726.2025.12.10.3.1

**Published:** 2025-12-01

**Authors:** Chris Moore, Mark Kingston, Idris Baker, Natasha Campling, Marika Hills, Emyr Jones, Sian Jones, Rashmi Kumar, Edward O’Brian, Alison Porter, Bernadette Sewell, Lauren Williams, Cendl Xanthe

**Affiliations:** Welsh Ambulance Services University NHS Trust ORCID iD: https://orcid.org/https://orcid.org/0000-0002-2192-3002; Swansea University Medical School ORCID iD: https://orcid.org/0000-0003-2242-4210; Swansea Bay University Health Board ORCID iD: https://orcid.org/0000-0002-9957-237X; University of Southampton ORCID iD: https://orcid.org/0000-0002-4158-7894; Dorothy House Hospice Care; Cardiff and Vale University Health Board ORCID iD: https://orcid.org/0000-0002-5577-7453; Patient and Public Contributor, Swansea University; Patient and Public Contributor, Swansea University; Welsh Ambulance Services University NHS Trust ORCID iD: https://orcid.org/0000-0002-9978-5025; Swansea University Medical School ORCID iD: https://orcid.org/0000-0002-3408-7007; Swansea Centre for Health Economics, Swansea University ORCID iD: https://orcid.org/0000-0001-5471-922X; Welsh Ambulance Services University NHS Trust ORCID iD: https://orcid.org/0009-0007-8880-6971; Welsh Ambulance Services University NHS Trust ORCID iD: https://orcid.org/0009-0005-2547-3028

**Keywords:** emergency medical services, end-of-life care, palliative care, pre-hospital care, primary health care

## Abstract

**Introduction:**

At the end of life, anticipatory or just-in-case (JIC) medications may help manage patients’ symptoms. Sometimes, emergency ambulances attend patients for whom JIC medications have not been prescribed. In Wales, UK, a Welsh Ambulance Services University NHS Trust (WAST) JIC intervention was launched in May 2020 in response to COVID-19, to enable ambulance paramedics to administer JIC medications to patients for whom they had not previously been prescribed. The ambulance JIC intervention is an ongoing feature of WAST pre-hospital care but has received limited evaluation. This study will explore the rationale, usage, costs and views of stakeholders of the WAST JIC medications intervention.

**Methods:**

We will employ a multi-method observational study design that incorporates both quantitative and qualitative aspects, informed by implementation science. We will prepare a detailed description of the WAST JIC medications intervention, its rationale and its use. We will interview paramedics and doctors who have provided the intervention, as well as paid and informal carers who were present during the care episode. We will also hold a focus group with paramedics who have not administered the intervention and undertake a cost analysis to estimate costs and savings associated with the intervention. We will use descriptive statistics to analyse quantitative data and a framework approach for qualitative data.

**Conclusion:**

This study, which focuses on the voices of patient advocates and practitioners, has the potential to shape future provision of this and similar services in WAST and other care providers.

## Introduction

End-of-life care (EoLC) in the community is a complex, multi-disciplinary area of healthcare, predominantly provided by primary and palliative care teams. Following recognition that a patient has reached their last year of life, anticipatory or just-in-case (JIC) medications may be prescribed and made available in the patient’s home, for use by community/palliative care clinicians, paramedics or trained informal carers. These injectable drugs are prescribed to ease symptoms such as breathlessness, anxiety or pain. They can also support efforts to enable people to remain and die at home if preferred (National Institute for Health and Care Excellence (NICE), 2015), and avoid unnecessary hospital conveyance and admission. The COVID-19 pandemic prompted the rapid introduction of new models of care, often with unknown or unmeasured benefits (Mitchell et al., 2021). One such intervention was the introduction of ambulance-based JIC medications to Welsh Ambulance Services NHS Trust (WAST) vehicles in May 2020. While implemented with COVID-19 patients in mind, these medications were also available to support symptom relief for others with an acute need at the end of life. Since the pandemic, the initiative has continued but remains under review, with the need for further evidence.

The WAST JIC initiative enables paramedics to administer medications to patients in their last days of life if home-based JIC medications are unavailable. To meet legislative and regulatory requirements, this is subject to a shared decision-making process with primary care or palliative care doctors. If in agreement that there is a need, the doctor will authorise the drug administration via a telephone verbal order (Wakefield & Wakefield, 2009). Further details of this process are outlined elsewhere (OʼBrian et al., 2023). The list of JIC drugs is summarised in Table 1.

**Table 1.** List of WAST JIC medications and related symptoms.

Medication	For the management of
Midazolam 10 mg / 2 ml	Anxiety, agitation
Haloperidol 5 mg/ml	Nausea/vomiting, delirium, terminal agitation
Glycopyronium 200 mcg/ml	Respiratory secretions (replaced by hyoscine hydrobromide during summer 2021)
Levomepromazine 25 mg/ml	Nausea/vomiting, delirium, agitation
Hyoscine hydrobromide 400 mcg/ml	Respiratory secretions

Morphine is not included in the above table, because it is a standard drug item, carried on all WAST emergency vehicles.

WAST was the first UK emergency ambulance service to introduce JIC medications; it is crucial to better understand the impact of this intervention on the ambulance service, its staff, and patients and carers. This is consistent with calls for further evidence around the provision and quality of EoLC in the community (Lord et al., 2012; McCormack et al., 2021; McGinley et al., 2017; Waldrop et al., 2015). Current literature is weighted towards specialist palliative care teams, social care workers (Briggs et al., 2021), community nurses (Mitchell et al., 2021) and patients’/relativesʼ experiences (Hanna et al., 2021). While recognition of the role paramedics could provide in EoLC is growing (Boughey, 2021; Juhrmann et al., 2022; Pentaris & Mehmet, 2019; Singer, 2021), little is known about the qualitative experiences of providing this care (Rees et al., 2021). Understanding the attitudes of clinicians and carers could lead to a more holistic understanding of system successes and failings, leading to better practice and adherence to patient wishes and outcomes (Hanna et al., 2021; McCormack et al., 2021).

There are some data highlighting paramedics’ concerns about the delivery of EoLC, related to inadequate training (McGinley et al., 2017; Rees et al., 2021), litigation worries (McGinley et al., 2017), ethical, moral and religious dilemmas (Leibold et al., 2018), difficulties accessing support networks out of hours (Hoare et al., 2018), conflict between family members and knowledge of patient care pathways (Juhrmann et al., 2022). A recent online survey of 920 UK paramedics’ experiences of EoLC found issues with access to advice, referrals, medical histories, care plans and medications (Campling et al., 2024). Understanding the views and experiences of paramedics providing EoLC could lead to policy and guidance that encourages confidence in practitioners to deliver care (Pentaris & Mehmet, 2019). Currently, evidence addressing clinicians’ perspectives on the rapid policy changes that occurred during the pandemic regarding EoLC in the community is limited (Mitchell et al., 2021). There is an opportunity to assess the uptake and use of the JIC initiative through analysis of routine data to help understand issues in implementation. This, in turn, can support intervention-related learning within and beyond Wales and can contribute to a broader understanding of the role of paramedics in EoLC.

### Aim and objectives

This project aims to address evidence gaps by exploring the rationale, use, costs and views of stakeholders of the WAST ambulance JIC medications initiative. Our objectives are to:

1. Describe the WAST JIC medications initiative, its rationale and its use.
2. Explore the views and experiences of paramedics, doctors and informal and paid carers.
3. Estimate the service-delivery costs and outcomes in relation to hospital avoidance.

## Methods

### Design

The WAST JIC medications initiative has been introduced into a complex real-world setting, with many actors and interactions. Implementation of an intervention is not straightforward and is subject to a range of influencing factors that depend on place, time and circumstances (i.e. the context in which the intervention is situated). This perspective is in line with the latest MRC framework for the development and evaluation of complex interventions, which emphasises ‘the value of understanding interventions as “events in systems” that produce effects through interactions with features of the contexts in which they are implemented’ (Skivington et al., 2021).

We propose to account for this complexity and multiplicity of stakeholders by using normalisation process theory (NPT) (May et al., 2009) as a conceptual tool to examine and explore the implementation of the JIC initiative. NPT is concerned with understanding how ‒ or if ‒ interventions become embedded or ‘normalised’ (i.e. enter routine use within the context studied). NPT characterises implementation in relation to the work undertaken by a range of actors across four domains:

- How people understand the innovation and its purpose (coherence).
- What decisions are taken to use the innovation (cognitive participation).
- What people do to bring the innovation into everyday use (collective action).
- How an innovation is reviewed, modified or abandoned (reflexive monitoring).

The research team has considerable experience in using NPT in a wide range of studies (Duke et al., 2020; Evans et al., 2022; Porter et al., 2016). We will use NPT in designing data collection instruments, including qualitative interview guides, as well as in our analysis, synthesis and writing. We will further exploit implementation science by using the theoretical framework of acceptability (TFA) (Sekhon et al., 2017). The TFA positions acceptability as a multi-faceted construct that reflects the extent to which people delivering or receiving a healthcare intervention consider it to be appropriate. The use of the TFA will complement the NPT framework as we explore multi-stakeholder perspectives on the JIC initiative, allowing us to expand on and develop our thinking, particularly in relation to the NPT domains of cognitive participation and reflexive monitoring.

**Figure 1:**
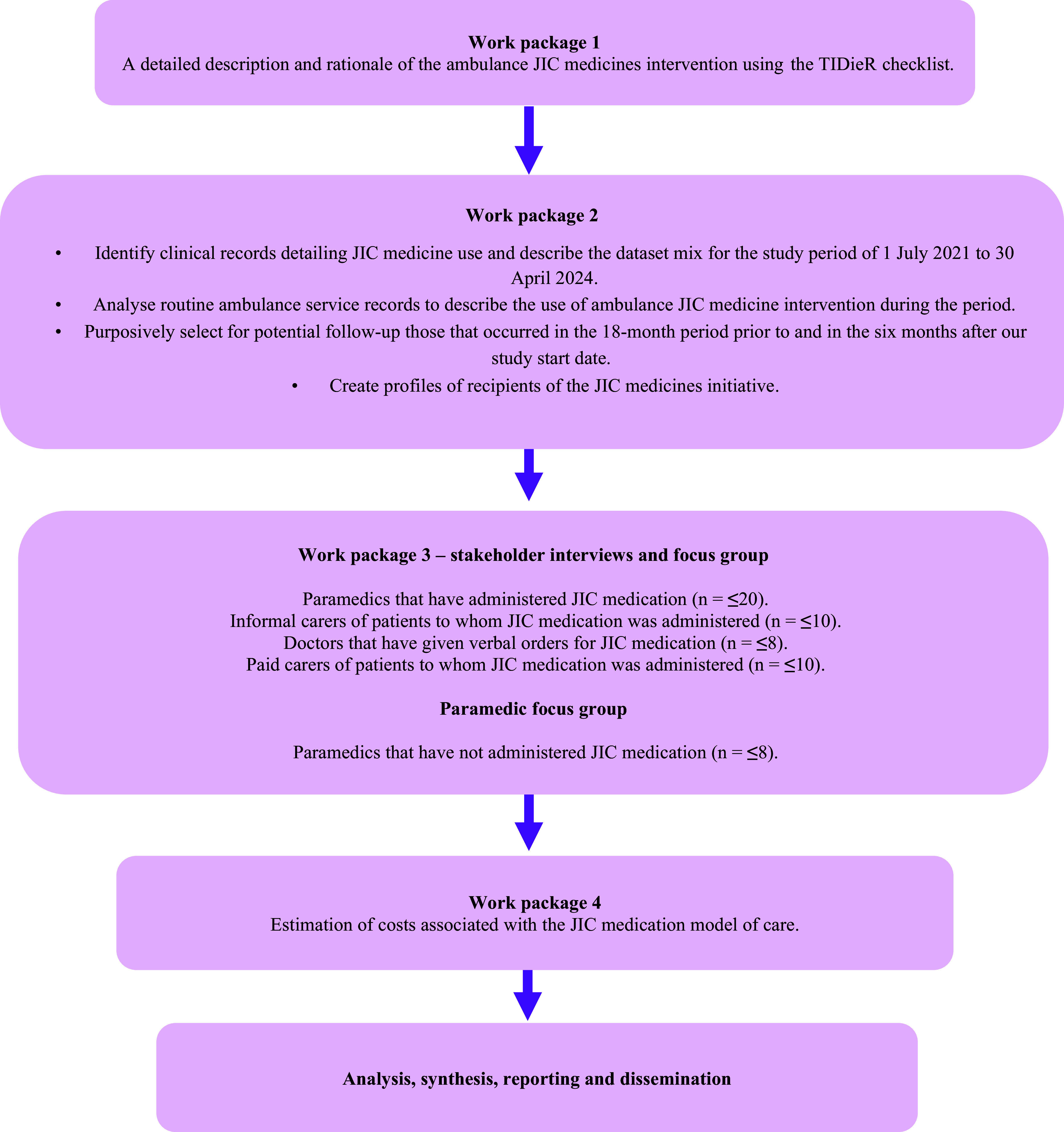
Figure 1. Study overview.

RELIEF is a multi-method study with four work packages (Figure 1). We will describe the WAST JIC medications initiative, interview paramedics, doctors, paid carers and informal carers, and hold a focus group with paramedics who have not administered JIC medications in EoLC. We will analyse WAST electronic patient clinical records (EPCRs) to identify ambulance JIC medication use and to collect incident response data, which will inform statistical and health economic analyses.

### Patient and public involvement

We are firmly committed to involving patients and the public, and will follow the UK Standards for Public Involvement (UK Public Involvement Standards Development Partnership, 2019). In developing this study, we presented and discussed the concept with a patient involvement group (Evans et al., 2020). Two patient/public contributors (RK/SJ) are co-applicants and sit on the research management group. Both have lived experience of supporting elderly parents in end-of-life care and terminal illness. They will be involved in and strengthen many aspects of the study, including the development of participant information materials, qualitative analysis and dissemination, including lay summaries.

### Setting

The study will be conducted in Wales, UK, with paramedics, doctors and paid and informal carers.

### Participant eligibility

Inclusion criteria for participants will be as follows:

- WAST paramedics who have administered WAST JIC medications (interview)
- WAST paramedics who have not administered WAST JIC medications (focus group)
- Doctors who have given verbal orders to WAST paramedics for the administration of WAST JIC medications (interview)
- Paid carers of patients to whom WAST JIC medications have been administered (interview)
- Informal carers of patients to whom WAST JIC medications have been administered (interview).

### Sampling

We will use WAST clinical records from 7 January 2021 to at least 30 April 2024 to identify and describe the use of the JIC medications initiative. To identify potential participants for qualitative data collection we will use a 24-month dataset, including records completed from 18 months before the study start date (1 October 2023) and six months after it, where a WAST JIC medication use has been recorded. Participant selection will start with the most recent relevant care episode and work backwards, with consideration for diversity and the geographical spread of participants.

### Work package 1: description of ambulance JIC medications initiative in Wales

The study team will create a detailed description and rationale of the WAST JIC medicines initiative by undertaking document analysis of relevant texts (Bowen, 2009; Moilanen et al., 2022). These include business cases, protocols, standard operating procedures and records of meetings. The description will include:

- Rationale
- Involvement of stakeholders (palliative care, pharmacy, WAST, primary care, paramedics)
- Choice of medications (doses, quantities)
- Processes and procedures to support safe drug administration
- The model of patient assessment by paramedics and consultation with the prescribing doctor.

We will use the Template for Intervention Description and Replication (TIDieR) checklist to structure our description (Cotterill et al., 2018; Hoffmann et al., 2014).

### Work package 2: ambulance service records analysis

#### Data collection

We will use quantitative methods to analyse up to 34 months of retrospective ambulance case records, which include the use of ambulance JIC medicines by paramedics. We will review WAST patient clinical records (PCRs) that document the administration of a WAST JIC medication or a patient’s own (home-based) JIC medication. We will also include a ‘narrative section’ search of PCRs to identify JIC medication uses that are not recorded in the medications section. For all patients with a JIC administration, we will report demographics and clinical characteristics (case-mix), response details such as job-cycle time, location (e.g. private home, nursing/care home), locality/area, resource deployed (ambulance / rapid response vehicle), episode duration, JIC medication administered and verbal order record submitted by the doctor. We will determine the distance to the nearest emergency department (ED) to estimate distance, travel time, fuel costs and, if possible, CO_2_ emissions.

### Work package 3: qualitative interviews and focus groups

The research team, including PPI partners, will prepare semi-structured interview guides for paramedics, doctors, informal carers and paid carers. Informed by the NPT and the TFA, the guides for clinical staff will explore factors that may support or hinder the initiative’s adoption, including its acceptability to stakeholders. The guides for informal and paid carers will examine perspectives on access to medications at the end of life and perceived benefits or drawbacks of the initiative. Data collection will be undertaken by study team members, with all interviews / focus groups recorded and transcribed in full.

To support the engagement of paramedic participants, paid carers and informal carers, we will offer a £25 voucher incentive. To support engagement of doctors, we will reimburse them for an hour of their time (at a rate of £80). We will offer informal carers the option to have an additional family member / friend present.

We have prepared distress protocols to support the needs of interviewees and interviewers (Draucker et al., 2009; McCosker et al., 2001). We will offer to pause or stop interviews/recording if necessary. We will provide clear messaging and information on access to psychological and bereavement support at the beginning and end of each interview. We will offer written materials to signpost participants to additional local support services and arrange a convenient time to telephone them (48 hours or so later), to check on the interviewee. We will also ensure our interviewers can debrief with an experienced research team member after all interviews.

#### Paramedics

We aim to interview up to 20 paramedics with recent experience of administering WAST JIC medications, identified from the WAST clinical records. WAST staff will identify potential paramedic participants from the clinical records and email them study information. We will invite paramedics with the most recent experience of a JIC medication administration (up to a maximum of 18 months), using purposive sampling to ensure demographic mix (age, sex) and geographical spread of participants.

We will prepare and circulate publicity materials within WAST, describing the aims and objectives of our study, to raise awareness among the paramedic population and to invite volunteers to a focus group with paramedics who have not administered an ambulance JIC medication. We will invite up to eight paramedics to a focus group, allowing us to compare their perceptions of the intervention with the experiences of paramedics who have administered ambulance JIC medications.

#### Doctors

We will use the 24-month dataset of WAST clinical records to identify doctors (GP / out of hours / palliative care), who have provided a verbal order for the administration of WAST JIC medications. We will interview up to eight doctors with direct involvement in the care episode that resulted in a verbal order for ambulance JIC medications being used.

#### Paid carers

We will work with the Enabling Research in Care Homes (ENRICH) Cymru group to plan and conduct our research with paid carers. ENRICH is a pan-Wales research network supporting study teams in work within the care home sector. ENRICH will review study materials and support care home engagement.

We will interview up to 10 paid carers, from residential care settings, who have been involved in the care of someone who was administered a WAST JIC medication. For incidents that are known to have occurred in a care home setting, we will initially contact the care home manager. This allows the manager to establish the identity of the paid carer who may have been present and to make a first approach as an independent and trusted colleague. If the paid carer expresses a willingness to participate, we will offer study information and a named contact for further details and to arrange an interview.

#### Informal carers

We will interview up to 10 informal carers (those providing unpaid long-term support to a person in need of care (e.g. family/friend (Lindt et al., 2020)), and who have been involved in the care of someone who was administered a WAST JIC medication).

WAST clinical records often include contact details (name and telephone) of close relatives. For incidents known to have taken place at a patient’s own or family home, at least three months after the date of death, we will use the details to send a sensitively worded participant information sheet outlining the aims of our study, as well as a consent to be contacted letter to read and return if they are interested in discussing participation.

For deceased persons who resided in care homes, we will approach care home managers and ask if they would be willing to make an initial approach to the bereaved, asking permission to send them study information. For deceased persons not in care homes, we will work with partners to develop an appropriate, sensitively worded information pack for relatives. We will limit attempts to establish contact to a single communication.

#### Data analysis

Quantitative data analysis will be undertaken using descriptive statistics, including measures of central tendency and variability (standard deviation / variance). Where appropriate, 95% confidence intervals will be used.

Qualitative outputs will be analysed thematically, using a reflexive and broadly inductive approach (Braun & Clarke, 2021). We will follow a framework analysis approach, which employs a structured method to analyse the data and which is suitable for a collaborative approach to analysis by a multi-disciplinary team to generate policy-relevant evidence (Gale et al., 2013; Ritchie et al., 2013). A qualitative analysis sub-group will be formed, led by qualitative lead AP and including clinicians and PPI members. Members of the team will familiarise themselves with the transcripts of interviews and focus groups, and devise codes drawn from these. An initial analytical framework will be agreed upon by the analysis sub-group, drawing on both data sets (patients and health care providers), then sub-group members will chart data onto the framework.

Each transcript will be read by a minimum of two members of the research team. The qualitative sub-group will discuss interpretation and emerging themes and consider any contradictions or inconsistencies. Analysis will take place first within groups (health professionals and patients) and then across the groups to explore triangulation opportunities. The subgroup will meet regularly to review progress with analysis and to discuss emerging findings. Findings will be written up in a structured manner around themes, with verbatim quotations used for illustration.

### Work package 4: estimation of costs associated with WAST JIC medications initiative

We will undertake a cost analysis to estimate the service-delivery costs and outcomes (in terms of potential cost savings in relation to hospital avoidance). This will include:

- Implementation costs (including costs of medications, paramedic and clinician time involved, ambulance costs and duration of care episode)
- Admission costs avoided (including costs of transport, emergency department attendance, hospital admission / bed day use).

In the early months of the study, we will develop a statistical and health economics analysis plan (SHEAP), to formalise and specify our data and analysis. The cost analysis will be based on an established approach (OʼBrian et al., 2023). We will use 30 months of retrospective ambulance case records to determine data on WAST resource use. We will estimate intervention costs, including JIC medications purchased/used/expired, paramedic and clinician time involved, call duration and transport costs.

We will also estimate costs of usual care (conveyance to hospital), including transport costs based on distance from nearest emergency department (ED), ED attendance costs and costs of hospital stay. Standard unit costs (e.g. personal social services, NHS reference costs) will be used to cost the resource use data, where available (NHS England, 2025; Personal Social Services Research Unit (PSSRU), 2023). Local finance data may need to be reviewed for costs that may be unavailable from standard sources. A net monetary benefit calculation will be undertaken to compare the JIC service costs with the cost of usual care from an NHS and personal social services perspective over the 30-month study time horizon. A standard discount rate of 3.5% will be applied where the time horizon exceeds 12 months. A deterministic sensitivity analysis will be conducted to address data uncertainty and provide a range of net monetary benefits for the JIC medication service.

### Data synthesis

We will formally synthesise the quantitative and qualitative analysis results, sequentially, using a triangulation protocol as described by O’Cathain et al. (2010) and the analytical approach outlined by Östlund et al. (2011).

### Ethics and dissemination

This study has been ethically approved (24 July 2024) by Wales REC 6, reference 24/WA/0179. We recognise the challenges and sensitivities related to this topic area, and particularly to engaging with bereaved relatives and carers. However, their lived experiences offer valuable and powerful insights that may shape the future delivery of pre-hospital EoLC. We will be informed by our public contributors, by published best practice and by consultation with NHS and independent sector bereavement providers, to ensure our processes and communications are appropriate and considerate.

We will maximise opportunities to publish our work and its outputs through academic, NHS and patient and public channels. To develop dissemination plans and outputs we will engage with service user, patient and professional groups, ambulance service networks (Association of Ambulance Chief Executives, National Ambulance Services Medical Directors, National Ambulance Research Steering Group, 999 Emergency Medical Services (EMS) Research Forum), primary and secondary care providers, care home networks including ENRICH, and NHS and third-sector palliative care providers.

## Conclusion

We believe this is an important and timely study, which incorporates multiple stakeholder perspectives on a complex intervention, including the voices of patient advocates, paramedics, doctors and carers. We examine an under-researched area of palliative care in the context of the developing role of emergency ambulance services. Results have the potential to identify gaps and barriers and inform better practices in the care provided by ambulance services and other providers.

## Acknowledgements

The RELIEF study team would like to thank the following for their support and assistance: ENRICH Cymru (Deborah Morgan, Leanne Brake); general practice advisor Rachel Lee; Health and Care Research Wales; the R&D team at WAST, notably Nigel Rees, Dmitri Holloway and Carla Jones; WAST sponsor Andy Swinburn, and the WAST clinical audit, informatics and information governance teams.

## Author contributions

CM, AP, MK, EJ, IB, EO’B, MH, NC, SJ, RK and BS are responsible for the conceptualisation of this study. CM, AP, MK, EO’B, NC, BS and CX are responsible for methodology and MK and LW for visualisation. Writing of the original draft was carried out by CM, MK and AP. Writing of the review draft and further editing was undertaken by NC, EJ, LW and CX. Project administration was undertaken by LW and MK. MK and CM contributed equally to this article as lead authors. MK acts as the guarantor for this article.

## Conflict of interest

None declared.

## Funding

This work is supported by a grant from the Health and Care Research Wales Research for Patient and Public Benefit programme (RfPPB) (reference: 1913).

## Statement of generative AI in scientific writing

The authors did not use a generative artificial intelligence (AI) tool or service to assist with preparation or editing of this work.
